# Estimating the Prognosis of Low-Grade Glioma with Gene Attention Using Multi-Omics and Multi-Modal Schemes

**DOI:** 10.3390/biology11101462

**Published:** 2022-10-05

**Authors:** Sanghyuk Roy Choi, Minhyeok Lee

**Affiliations:** School of Electrical and Electronics Engineering, Chung-Ang University, Seoul 06974, Korea

**Keywords:** prognosis estimation, low-grade glioma, deep learning, gene expression, prognostic gene, ensemble learning, attention mechanism

## Abstract

**Simple Summary:**

The estimation of the prognosis of low-grade glioma (LGG) patients using deep learning models and gene expression data has been intensively studied in recent years. Existing studies, however, have only considered mRNA expression data, ignoring other expression data and clinical data. The Multi-Prognosis Estimation Network (Multi-PEN), a deep learning model that employs multi-omics and multi-modal schemes, is proposed in this study to address this limitation. Using Multi-PEN, MYBL1 and hsa-mir-421 were identified as the most significant mRNA and miRNA, respectively, in the prognosis of LGG patients. Existing studies that estimate prognostic mRNAs and miRNAs support the findings of this study.

**Abstract:**

The prognosis estimation of low-grade glioma (LGG) patients with deep learning models using gene expression data has been extensively studied in recent years. However, the deep learning models used in these studies do not utilize the latest deep learning techniques, such as residual learning and ensemble learning. To address this limitation, in this study, a deep learning model using multi-omics and multi-modal schemes, namely the Multi-Prognosis Estimation Network (Multi-PEN), is proposed. When using Multi-PEN, gene attention layers are employed for each datatype, including mRNA and miRNA, thereby allowing us to identify prognostic genes. Additionally, recent developments in deep learning, such as residual learning and layer normalization, are utilized. As a result, Multi-PEN demonstrates competitive performance compared to conventional models for prognosis estimation. Furthermore, the most significant prognostic mRNA and miRNA were identified using the attention layers in Multi-PEN. For instance, MYBL1 was identified as the most significant prognostic mRNA. Such a result accords with the findings in existing studies that have demonstrated that MYBL1 regulates cell survival, proliferation, and differentiation. Additionally, hsa-mir-421 was identified as the most significant prognostic miRNA, and it has been extensively reported that hsa-mir-421 is highly associated with various cancers. These results indicate that the estimations of Multi-PEN are valid and reliable and showcase Multi-PEN’s capacity to present hypotheses regarding prognostic mRNAs and miRNAs.

## 1. Introduction

Owing to the rapid development of deep learning models, artificial intelligence based on deep learning has dominated various fields [1,2,3]. Deep learning models are being used extensively in diagnostics [4,5,6], medical imaging [7,8,9,10], and genome sequencing [11,12,13]. Additionally, the prognosis of various diseases can be estimated by deep learning models [14,15,16]. In these models, clinical variables, such as age and sex, are often utilized to predict prognosis. This study aims to propose a deep learning model for the estimation of the prognosis of low-grade glioma (LGG) [17]. Tumors in the central nervous system are defined by their origin and their histopathological features [18]. Gliomas are neuroepithelial tumors stemming from the supporting glial cells located in the central nervous system.

LGG is a group of primary brain tumors frequently generated in young and healthy patients. LGG consists of grade one tumors and grade two tumors. Grade I LGG tumors do not involve histologic features such as atypia, mitotic activity, or necrosis. In contrast, grade II LGG tumors do involve the aforementioned histologic features [19]. Symptoms of LGG include headaches, vomiting, blurry vision, memory loss, nausea, and weakness on one side of the body [20,21,22,23]. LGG causes seizures more frequently than other brain cancers because it is produced in the cerebral cortex. Generally, LGG patients show a better prognosis compared to high-grade glioma (HGG) patients. However, if it progresses to HGG, the prognosis may drastically deteriorate, eventually leading to death [24]. As a result, the early diagnosis and treatment of LGG are essential in order to increase the likelihood of survival.

Gene expression has become remarkably significant in the identification of tumors. For instance, mutations of isocitrate dehydrogenase 1 and 2 (IDH1 and IDH2) act as tumor markers [19]. Additionally, the capicua transcriptional repressor (CIC) gene on chromosome 19q has recently been extensively explored, where oligodendroglioma and oligoastrocytomas cooperate with the mutation of the CIC gene [25,26]. CIC gene mutations are discovered in 69% of oligodendrogliomas. Additionally, mutations of IDH1 and IDH2 and 1p/19q codeletion have been proven to collaborate with CIC gene mutations. Moreover, the overexpression and mutation of tumor protein 53 (TP53) are described as a genetic feature of gemistocytic astrocytoma and low-grade astrocytoma.

Therefore, the relationship between gene expression and prognosis in LGG has been widely studied to estimate the prognosis with deep learning models using gene expression. For instance, the Gene Attention Ensemble NETwork (GAENET) was proposed as a deep learning model to estimate the prognosis of LGG patients [27]. In GAENET, the gene attention mechanism, which is a modification of the attention mechanism [28,29,30], was introduced. The gene attention layer scores genes with respect to prognosis. The value of the scores ranges from zero to one, in which a high value indicates a close relationship with the prognosis. Since the gene attention layer is trained by a deep learning process, nonlinear relationships are studied to select the prognostic genes, which is difficult to do in other conventional models. Additionally, residual learning [31] and ensemble learning methods [32] are employed in GAENET, which uses deep learning methods to enhance the prediction performance.

Numerous studies have demonstrated that using multi-omics and multi-modal data results in improved performance [33,34,35]. For example, a study proposed two models using multi-omics data: a risk score model that estimated the prognosis of LGG patients based on six specific genes and a radiomic risk score model that exploited magnetic resonance images to predict the pre-operation risk score [33]. Another study used a multivariate Cox-PH model with various data. It applied numerous combinations of clinical, radiomics, and molecular data [34]. An integrated multi-omics deep learning network method (i-Modern) uses one autoencoder to extract significant elements from multi-omics data [35].

Therefore, a crucial limitation of GAENET is that a limited transcriptome data type is used in the model. Specifically, GAENET only uses mRNA data, but other transcriptome data types are expected to be related to the prognosis. For instance, it is widely known that miRNA controls mRNA by regulating mRNA and hindering its expression [36]. Such a property signifies that miRNA has the potential to be highly related to the prognosis of LGG patients. Additionally, it is generally accepted that clinical data are also highly related to prognosis.

This paper proposes a deep learning model that employs not only mRNA data but also miRNA and clinical data. The motivation for this study comes from the idea that such a perspective with multi-omics and multi-modal schemes can provide a better understanding of the prognosis of LGG patients. Given this additional information relating to miRNA, the identified prognostic genes may be distinct from those identified when using GAENET and may explain the relationship between mRNA and miRNA in terms of patient prognosis. While there are several studies that take advantage of the high correlation between mRNA and miRNA [37,38], in this study, a multi-modal scheme [39] was additionally used with the clinical data. Moreover, via gene attention layers that use miRNA data and clinical data as inputs, it can be estimated which miRNA is most likely to be related to the prognosis of LGG.

## 2. Background

### 2.1. Gene Attention Layer

Deep learning models commonly consist of multiple layers. Each layer comprises an input, a weight matrix, and nonlinear activation functions. The layer receives input from the last layer and multiplies the input and weight matrix [40]. The values made from multiplying the input and output pass through the nonlinear activation function and are transferred to the subsequent layer. This process is repeatedly conducted until it reaches the output layer. This process can be represented by the following equation:

(1) $$\hat{Y}=\tau_{N}\;\left(W_{N}\cdot \tau_{N-1}\;\left(W_{N-1}\cdots \tau_{2}\;\left(W_{2}\cdot \tau_{1}\;\left(W_{1}\cdot X\right)\right)\right)\right),$$

where $\hat{Y}$ is an estimation vector that is the output of the deep learning model; $\tau_{k}$ and $W_{k}$ are the nonlinear function and weight matrix in the $k^{th}$ layer, respectively; X is an input vector of the deep learning model; and N is the total number of layers used in the model. Training a deep learning model is identical to finding an appropriate $W_{k}$ to estimate $\hat{Y}$ properly for a given $\hat{X}$.

The attention mechanism is a method of multiplying the output vectors of $\tau_{k}$ by specific values from zero to one. This method reduces the effects of features less related to the target. Thus, a lower value, approximately zero, indicates that the corresponding feature has a low probability of having a relationship with the target. This process can be considered a filter to eliminate less related features. The mechanism can be represented by the following equation:

(2) $$X_{k+1}\;=A^{T}⊙\tau_{k}\;\left(W_{k}\cdot X_{k}\right),$$

where $X_{k}$ is the output of the ${\left(k-1\right)}^{th}$ intermediate layer; A is an attention vector composed of the attention values of all input features of $X_{k}$; and the ⊙ operator denotes component-wise multiplication. The attention vector A is not a constant and is instead a deep learning module that learns and changes its value in every epoch based on the backpropagation in deep learning training.

The attention layer in GAENET uses a squeeze and excitation (SE) block composed of two fully connected networks. Thus, the block has two weight matrices, and the first weight matrix is multiplied by gene expression inputs; it reduces the dimension of the gene expression, resulting in feature extraction. Therefore, the first weight matrix has a dimension of q×p, where p is the number of genes and q≪p. The next layer, accordingly, has a dimension of p×q, producing an attention vector with p dimension. These processes can be represented as follows:

(3) $$A\;=\sigma \left(W_{A2}\cdot ReLU\left(W_{A1}\cdot X_{0}\right)\right),$$

(4) $$X_{1}=A^{T}⊙X_{0},$$

where σ denotes the sigmoid activation function; $W_{A1}$ and $W_{A2}$ indicate the first and second weight matrices of the attention layer, respectively; $X_{0}$∈$R^{p}$ is the gene expression input; and $X_{1}$∈$R^{p}$ is the output of the attention layer. Consequently, $X_{1}$ becomes the input of the deep learning model following the attention layer.

Owing to the attention vector A with values between zero and one, the input gene expression data are regulated before they are transferred to the subsequent deep learning layers. The attention layer assigns values close to 1.0 to highly target-related genes, whereas it assigns values close to 0.0 to the genes considered less related to the target. Two weight matrices in the attention layer are trained during the training process simultaneously with the other deep learning layers. This process shows that the attention value is determined via training on gene expression data and is not manual.

Therefore, the attention value of each gene tells us how important the gene is. Hence, the target-related genes can be discovered. In other words, the prognostic genes can be found by the attention layer when the prognosis is trained to be predicted by the model. Similar to other attention mechanisms, this gene attention layer also regulates deep learning models to avoid overfitting models, reducing overfitting problems caused by a lack of data.

### 2.2. Residual Learning

The vanishing gradient problem is a common problem for deep learning models that use many layers. The cause of the vanishing gradient problem is multiplied gradients in the chain rule. The backpropagation algorithm used to train deep learning models calculates the gradients of the initial layers via the chain rule, which involves multiplying multiple gradients. If the gradients are between zero and one, the multiplication of the gradients makes the gradients of the initial layers converge upon zero. Since the conventional training algorithm for deep learning models depends on the gradients, significantly low gradient values in the initial layers cause a training failure in the layers.

Residual learning with a residual network is a method to alleviate the vanishing gradient problem. Residual networks consist of a series of layers with a skip connection between the input of the first layer and the output of the last layer [31]. In this connection, the input is added to the output, resulting in a capability to simplify the multiplication in the chain rule. Since the vanishing gradient problem is caused by a series of multiplications in the chain rule, such a simplification can reduce the problem. Therefore, residual learning enables us to stack more layers compared to conventional deep learning models. Residual networks with two layers can be represented as follows:

(5) $$X_{k+2}=X_{k}+\tau_{k+1}\;\left(W_{k+1}\cdot \tau_{k}\;\left(W_{k}\cdot X_{k}\right)\right),$$

where $X_{k}$ is the output of the previous layer.

### 2.3. Ensemble Learning

Ensemble learning is a method to prevent deep learning models from overfitting by using multiple models on the same target [32,41]. Due to the fact that it has more parameters than samples in general, deep learning can result in overfitting issues. Such deep learning models with overfitting issues make accurate predictions from the training set, while the test set accuracy declines. In order to find a solution to the overfitting issue, the method of ensemble learning is utilized. In ensemble learning, each of a number of modules acquires new knowledge from the data. Then, the next step is to select the most effective modules to demonstrate the entire model or to use the result that represents a weighted sum of all of the modules as the final result. The model can also be protected from outliers, which can be removed by averaging the results of multiple learning. Moreover, ensemble learning can address problems caused by the random selection of inputs for mini-batch sampling. If outliers are included in the training set for a mini-batch, a single model has a probability of diverging or learning inappropriate features. However, ensemble learning methods effectively circumvent the aforementioned difficulties with the effect of multiple modules.

In this study, a deep learning ensemble model with multiple modules is presented. Each module in the ensemble deep learning model employs the same structure. Due to the random initialization of weight parameters and mini-batch sampling, even when the same structure is employed, these modules generate distinct results. These diverse results can counteract the randomness of conventional deep learning training, in which the results vary. As the results of multiple modules are averaged in the ensemble deep learning model, this issue can be mitigated. The final prediction derived from the ensemble deep learning model is computed as follows:

(6) $$\hat{Y}\;=\frac{1}{n}\underset{i\in }{\sum }f_{i}\left(X\right)$$

where n is the number of modules in the ensemble deep learning and $f_{i}$ represents a module with an equivalent structure.

### 2.4. Layer Normalization

Layer normalization is a regularization method to maintain the output distributions of the layers [42]. During the training process of deep learning, the output distributions consistently fluctuate. This property of training can hinder the convergence of training since it can be interpreted that each layer learns different sample spaces in terms of the input of the layer. Thus, the optimal convergence point also fluctuates, resulting in the possibility of failure in training. To address this limitation, layer normalization is adopted in the proposed model. In the residual learning modules, layer normalization is employed before activation functions, which is the conventional way to use layer normalization.

### 2.5. The Gene Attention Ensemble Network

The GAENET, which is one of the baselines of the proposed model, employs mRNA to predict the prognosis of LGG patients. To handle mRNA data and extract the features regarding the prognosis, the attention layers are used as the first layer. Then, two residual modules are trained in the ensemble structure. In these modules, layer normalization and dropout are used. As a result of the prognosis estimation, the average outcome of these modules is computed. However, in GAENET, mRNA data are employed, which can be one of the limitations since deep learning training with different data types can consider different aspects of the prognosis of LGG. This limitation motivated us to explore a novel method of using multi-omics and multi-modal schemes integrated with a deep learning model.

### 2.6. Baselines

Four conventional machine learning models to estimate survival times are employed as baselines: ridge regression, survival support vector machine (SurvivalSVM) [43], random survival forest (RSF) [44], and Coxnet [45]. Ridge regression is a method that can be utilized to analyze data that have multicollinearity or to estimate a prediction vector. This method exploits L2 regularization and can handle the problem in conventional linear regression wherein the prediction vectors in multiple regression are not orthogonal, so parameter estimation based on the minimum sum of squares is likely to be poor. Coxnet is a regularized Cox proportional hazard with L1, L2, and both L1 and L2. A random forest is a probabilistic predictor that uses a mean to improve prediction accuracy and drops over-fitting when fitting several decision tree classifiers. A support vector machine is one of the supervised machine learning models. Support vector machines are commonly used in classification tasks to reduce the dimensions of the dataset.

## 3. Methods

### 3.1. Multi-Omics and Multi-Modal Ensemble Deep Learning

Numerous functions of RNAs have been discovered and studied as a result of technological advances in genome sequencing. It has been widely accepted that mRNA is highly correlated with miRNA, which regulates the function of mRNA and exerts a significant influence on diseases. Consequently, the model proposed in this paper, the Multi-Prognosis Estimation Network (Multi-PEN), utilizes multi-omics data consisting of mRNA and miRNA [46,47]. Two attention layers are utilized for multi-omics data, each of which identifies the prognostic genes and assigns a high attention value to significant genes. As was mentioned in the previous section, the learning of attention values is also performed by the backpropagation algorithm of deep learning, which can be interpreted as learning from data.

Additionally, clinical data, such as age at diagnosis and sex, are known to be associated with the prognosis of LGG, as many studies have aimed to predict the prognosis using such variables. In Multi-PEN, the clinical data are employed as one of the multi-modal inputs of the model. Since there are several categorical variables in the clinical data, they are encoded as one-hot vectors; since the computer machine is incapable of perceiving human language, clinical categorical variables must be encoded into vectors consisting of zero and one, of which an index of one indicates the status of the corresponding category, such as sex or features of tumors comprising clinical data. Then, all clinical variables are concatenated to a single vector. This clinical vector is also trained with an attention layer, the same as mRNA and miRNA datatypes.

**Figure 1:**
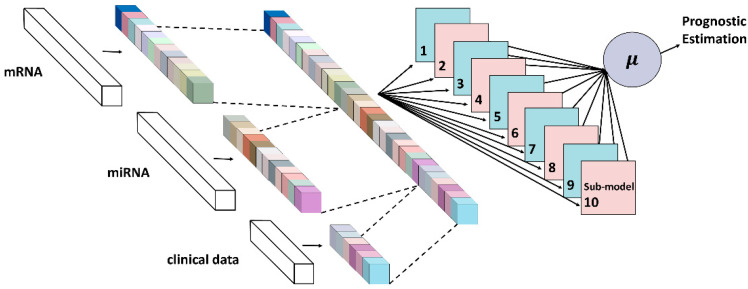
The deep learning architecture of Multi-PEN. The second layers with colors indicate the gene attention layers. The data multiplied by the attention values are transferred to sub-models. The estimation is the average value of each model.

Figure 1 depicts the structure of Multi-PEN. Three different datatypes with multi-omics and multi-modal schemes are employed as the inputs. These inputs are transferred to the attention layers and multiplied by the attention values that are learned with each data type. The attention values range from zero to one, where higher values indicate greater association with the target, i.e., the prognosis of LGG. Then, ten sub-models for deep ensemble learning take the inputs and predict the prognosis of LGG patients. While each prediction value is distinct among the sub-modules, the averaged values of these predictions can generalize the prediction and stabilize the random effects of deep learning.

**Figure 2:**
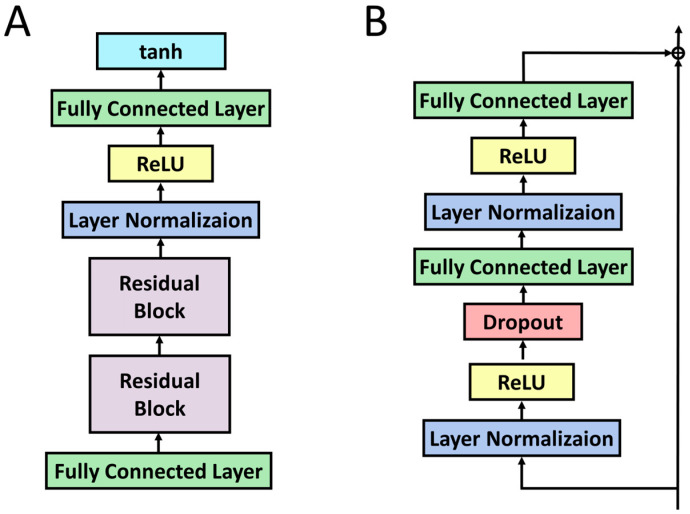
The detailed architecture of the sub-models in Multi-PEN. (**A**) The sub-model structures. (**B**) The structure of each residual block in the sub-models. The structure of the residual block in (**A**) is de-scribed in (**B**). The equivalent role organizing in (**A,B**) is colored identically.

Each sub-model consists of residual networks, as is shown in Figure 2A. Two residual blocks are used in the sub-models, each of which is composed of two fully connected layers, as is shown in Figure 2B. Layer normalization and the dropout technique are utilized between the layers to reduce the overfitting problem. The predictions of each sub-model are calculated with the hyperbolic tangent activation function, where the value ranges from −1.0 to 1.0. A higher value indicates a high likelihood of survival, similar to GAENET (because the same loss function is used).

### 3.2. Clinical Data

Multi-modal is used to refer to a framework that utilizes various datatypes and contexts. Multi-PEN employs a multi-modal framework with gene expression data and clinical data in order to enhance the estimation performance and to discover prognostic mRNA and miRNA while considering clinical data. Clinical variables used in the model are the age at diagnosis, sex, race, ethnicity, tumor grade, and the number of mutations. Isocitrate dehydrogenase (IDH) codel subtypes are also included in the variables since it has been posited that IDH1 and 2 are significant prognostic and therapeutic biomarkers for glioma. Furthermore, 1p19q codeletion is also used for the same reason [48]. These clinical data are preprocessed to be used in Multi-PEN. The preprocessing methods are described in Section 3.1.

### 3.3. TCGA Data—Low-Grade Glioma

To evaluate Multi-PEN, The Cancer Genome Atlas for Low-Grade Glioma (TCGA-LGG) dataset was used in this study. In this experiment, 125 uncensored samples are employed to perform five-fold cross-validation; among the samples, 100 samples are randomly selected to train the model, and the other samples become test sets in each cross-validation. This five-fold cross-validation is performed 20 times; therefore, 100 experiments with different training and test sets are conducted [49]. For the preprocessing process, if the zero value of a gene exceeds half of the samples, the gene is omitted from the dataset. Additionally, if the samples have a missing value, the corresponding genes are excluded. The gene expression data are preprocessed by log normalization.

### 3.4. K-Fold Cross-Validation

K-fold cross-validation is used in various domains, including the medical domain, in order to make a dataset similar to the conditions in clinical tests [49,50,51]. K-fold cross-validation is a principled method for dividing a dataset into a training dataset and a validation dataset. Then, a machine learning model trains only with the training dataset, without any information in the validation set, including the number of samples and value distributions of each variable. In K-fold cross-validation, the dataset is equally and randomly divided into *K* sub-datasets to give the same number of samples. In this division process, the sub-datasets do not share any information, which gives identical conditions to clinical tests; therefore, the normalization process and preprocessing of the data are conducted only with the training set after the division; the validation set is preprocessed with the information of the training set. Then, K−1 sub-datasets are used as the training set, and the other dataset is used as the validation set. This trial is repeated *K* times with different validation sets in each trial. Such processes of K-fold cross-validation enable a similar condition to *K* repetitions of clinical tests with different test sets.

### 3.5. Hyperparameter

In the experiment, Multi-PEN employs a total of ten sub-models. In the residual block, the number of nodes in the first and second fully connected layers is 16 and 8, respectively. Having such a small number of nodes in the fully connected layers is a result of the overfitting issue, where the number of samples is extremely small compared to the number of genes. The number of the attention layer nodes is set to 10 for each of the three attention layers. An optimization algorithm known as Adam is used, with the learning rate set to 0.0001. In this study, 20 epochs are used to train the model, and five mini-batches are organized in each epoch. For the performance comparison, the C-Index [52] is measured with the proposed model and other baseline models.

## 4. Results

**Figure 3:**
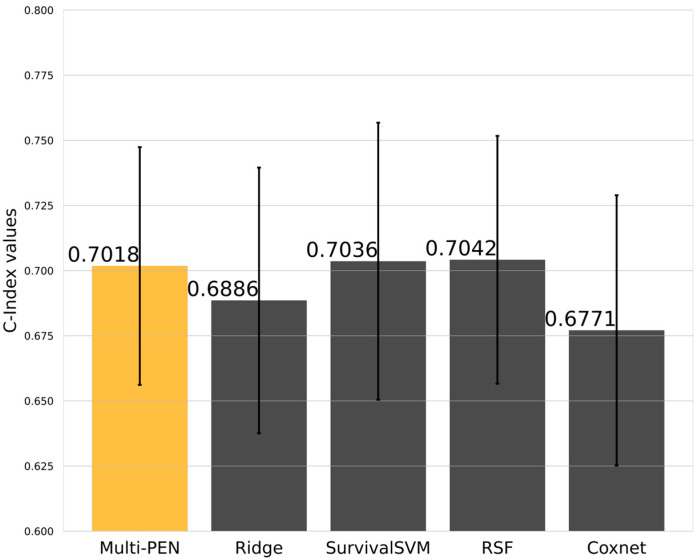
C-Index of Multi-PEN and other conventional models with 100 experiments.

A comparison of the performance measured by the C-Index of Multi-PEN, ridge regression, SurvivalSVM, RSF, and Coxnet is shown in Figure 3. With 20 times five-fold cross-validation, the C-Indices were evaluated 100 times. The C-Index of Multi-PEN was 0.7018. While RSF exhibited effectiveness in terms of performance, Multi-PEN outperformed ridge and Coxnet, for which the performance was 0.6886 and 0.6771, respectively. In summary, Multi-PEN demonstrated competitive performance with other conventional methods.

As shown in Figure 3, the performance differences between the models were marginal, where the best model and the worst model exhibited a 4.0% difference in terms of the C-Index. Although Multi-PEN did not display outstanding performance compared to the conventional models, the performance difference between the best model and Multi-PEN was 0.3%, which can be interpreted as a competitive performance. Since deep learning models generally demonstrate better performances as the number of training samples increases, it is expected that this limitation can be handled in future studies with expanded samples.

**Figure 4:**
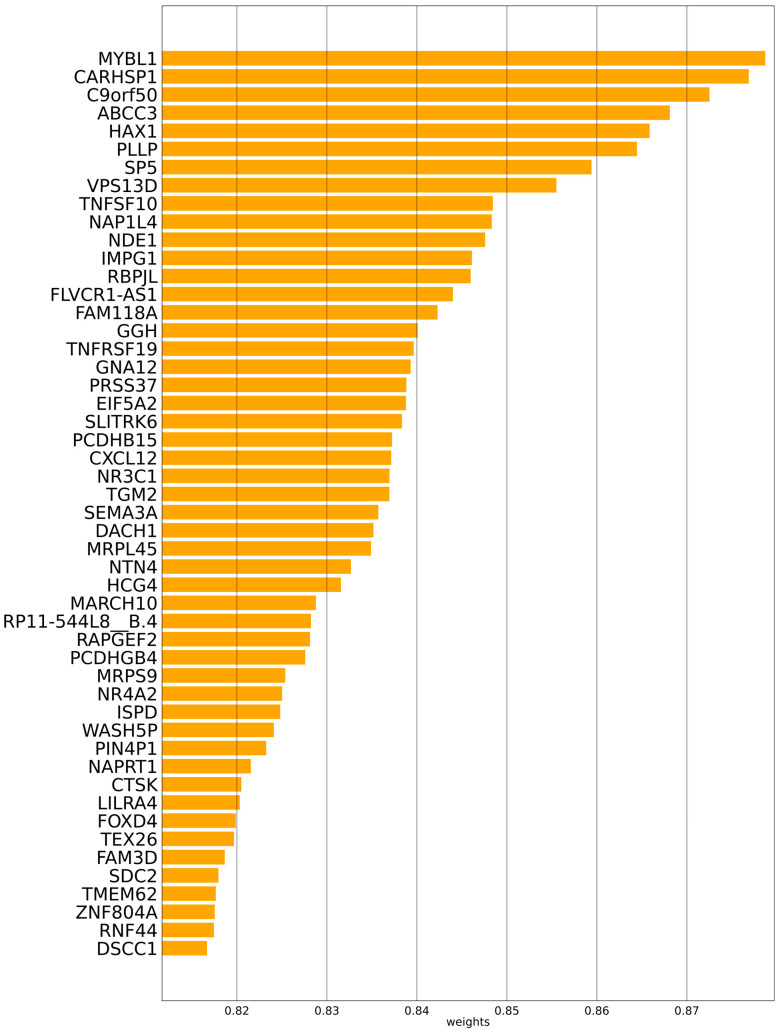
Estimated prognostic mRNAs by Multi-PEN and their attention values.

**Figure 5:**
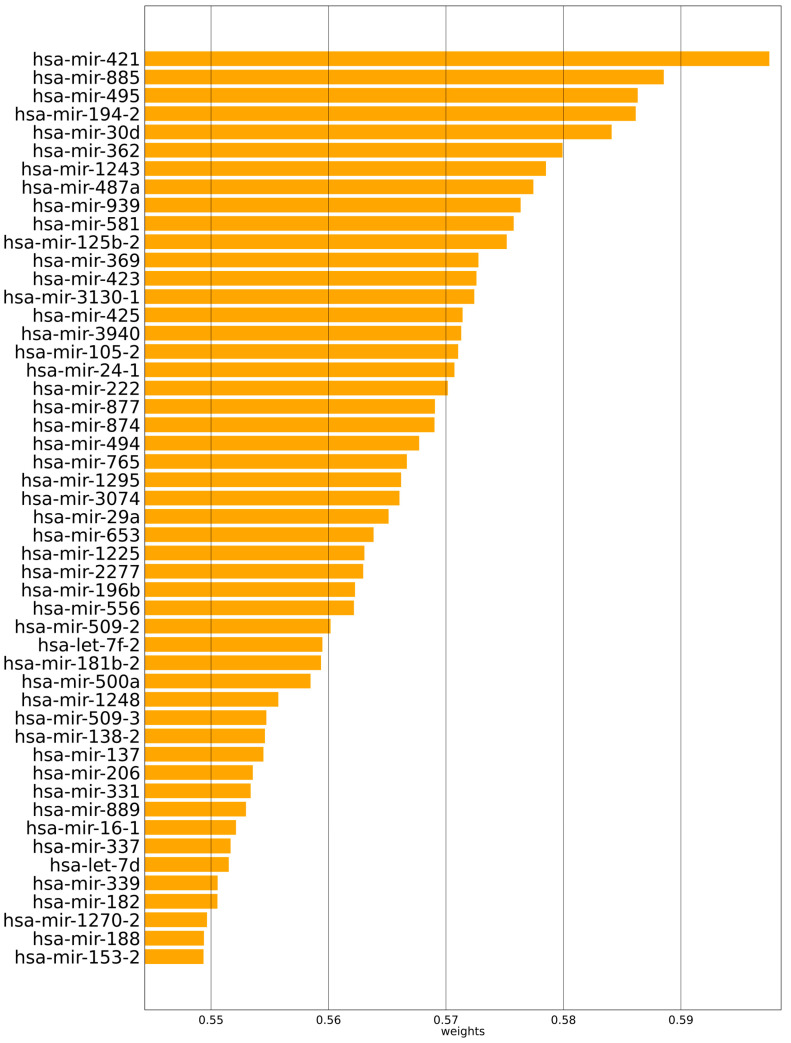
Estimated prognostic miRNAs by Multi-PEN and their attention values.

The prognostic ranks of mRNA and miRNA can be found in the gene data using Multi-PEN, which is one of the main advantages of the model. This can lead us to the discovery of prognostic mRNA and miRNA. The values of the attention layer, which are shown in Figure 4, are where the prognostic genes of mRNA are located. Figure 5 also displays the fifty miRNA genes that may be used to predict outcomes. MYBL1 was shown to be the mRNA with the highest attention value, followed by CARHSP1, C9orf50, ABCC3, and HAX1. In addition, the prognostic miRNAs that were discovered by Multi-PEN are as follows: hsa-mir-421, hsa-mir-885, hsa-mir-495, hsa-mir-194-2, and hsa-mir-30d.

## 5. Discussion

The proposed deep learning model, Multi-PEN, was used to estimate prognosis and search for prognostic genes in LGG patients. Other studies have utilized conventional and straightforward deep learning models, such as MLP, without considering current developments in deep learning [21,22]. Conversely, to find the prognostic genes, including miRNA, Multi-PEN exploits recent developments, i.e., residual networks and gene attention mechanisms. The prognostic mRNA and miRNA were estimated using the gene attention mechanism. The prognosis prediction performance of Multi-PEN was competitive with other models, which suggests that the estimated prognostic mRNA and miRNA are reliable.

This study proposed a novel way of investigating prognostic mRNA and miRNA from a nonlinear perspective, while other studies have focused on linear perspectives with the Cox proportional-hazards model and t-test. The estimation of the prognostic genes can be conducted by a deep-learning-based attention algorithm embedded in Multi-PEN. Because prognostic genes are evaluated with combinational effects and polynomial correlations, gene attention can be considered to find genes from a nonlinear perspective. In contrast, the Cox proportional-hazards model and Kaplan–Meier estimation, which are commonly used in a variety of studies, are focused on the linear relationship between genes and outcomes.

MYBL1 was estimated to be the most significant mRNA with a gene attention value of approximately 0.88. This indicates that MYBL1 has a probability of possessing a direct relationship with the prognosis of LGG. Such an estimation of Multi-PEN is in accordance with several existing studies that have suggested that MYBL1 regulates cell survival, proliferation, and differentiation [53], which are highly associated with tumorigenesis.

Additionally, CARHSP1 was identified as a significant prognostic mRNA with the second-highest attention value. This result is also related to a recent study [54] that investigated whether CARHSP1 is radiotherapy protective in glioblastoma by signaling via the CARHSP1/TNF-α pathway. In addition, patients with high levels of CARHSP1 who are treated with radiotherapy have a poor prognosis. It was reported that ABCC3, one of the other prognostic mRNAs estimated by Multi-PEN, has a connection with a poor prognosis and resistance to treatment in cancer [55]. Additionally, according to the study, the intracellular concentration and efficacy of drugs are decreased by the exporter that releases the drugs from within the cells; thus, overexpressed ABCC3 creates resistance to multiple drugs for cancers, which may affect the prognosis of LGG. Another estimated prognostic mRNA, HAX1, has been reported to be related to lung cancers; HAX1 was overexpressed in non-small cell lung cancer; however, it was found that HAX1 was not overexpressed in normal cells [56]. Additionally, the high concentration of HAX1 gene expression was related to the TNM stage, lymphatic metastasis, and tumor size. While HAX1 has been previously reported as being related to lung cancers, the estimation of Multi-PEN implies that the gene also has a probability of being associated with LGG. Conversely, among the top five prognostic mRNAs estimated by Multi-PEN, C9orf50 has not been extensively studied regarding its relationship with LGG or other cancers. Further studies should be conducted on the relationship between C9orf50 and LGG, since the gene has a high attention value according to Multi-PEN.

Furthermore, prognostic miRNAs were also identified by Multi-PEN, which considered the relationship with mRNAs and other miRNAs, resulting from the gene attention layers in Multi-PEN. As a result, hsa-mir-421 was estimated to be the most significant miRNA with an attention value of 0.60; other estimated prognostic miRNAs were hsa-mir-885, hsa-mir-495, hsa-mir-194-2, and hsa-mir-30d. These results accord with those of existing studies demonstrating the overexpression of hsa-mir-421 in a variety of cancers, including gastric cancer [57,58], neuroblastoma [59], lung adenocarcinoma [60], breast cancer [61], and osteosarcoma [62]. Additionally, a study showed that has-mir-421 was highly expressed in lung cancer cells [63]. Furthermore, it has been demonstrated that the overexpression of has-mir-421 is a prognostic biomarker for non-small cell lung cancer and encourages tumor growth [64]. Considering these findings from existing studies, the estimation results by Multi-PEN are supported.

Additionally, another prognostic miRNA estimated by Multi-PEN, hsa-mir-885-5p, was reported to be upregulated in colorectal cancer cells [65]. Accordingly, the suppression of has-mir-885 restrains tumor cells from proliferating or migrating. Furthermore, it is known that abnormal expression of has-mir-885-5p has a possibility of reconstructing hepatocellular carcinoma metabolism and progression [66]. The direct relationship between hsa-mir-885 and LGG has not yet been investigated; however, it can be considered that hsa-mir-885 is associated with LGG based on these existing studies on hsa-mir-885 expression in cancer cells. Another estimated miRNA, hsa-mir-495, has been known as a tumor-suppressor [67]. Additionally, it was found that has-mir-495 is an oncogene [68]. Another miRNA, hsa-mir-194, has been linked to tumor metastasis and medication resistance in non-small cell lung cancer (NSCLC); hsa-mir-194 levels declined in NSCLC samples when compared to non-cancerous lung tissues, and a low hsa-mir-194 expression was associated with a poor prognosis [69]. For hsa-mir-30d, high expression contributed to invasion, proliferation, and tumor growth, resulting in poor survival of prostate cancer patients [70]. In addition, hsa-mir-30d overexpression inhibits the activation of cyclin D1, cMyc, and catenin, three essential components of the Wnt/beta-catenin signaling pathway, thereby inhibiting the development of colorectal cancer [71]. In contrast, in pancreatic cancer, hsa-mir-30d was significantly downregulated compared to non-tumor tissues and cells [72]. The capability of Multi-PEN to identify prognostic genes is supported by these existing studies. In addition, potential prognostic genes for LGG have been identified in this study and can be investigated further in future research.

## 6. Conclusions

Multi-PEN, a deep learning model with multi-omics and multi-modal schemes, was proposed in this paper to estimate the prognosis of LGG and prognostic genes. The general structure of Multi-PEN is similar to that of GAENET; however, the attention layers for miRNA expression and clinical data were additionally employed in Multi-PEN. Owing to the advantage of the gene attention mechanism, it can be used to identify prognostic mRNA and miRNA.

As a result, several mRNAs were estimated as prognostic mRNAs, including MYBL1, CARHSP1, C9orf50, ABCC3, and HAX1. Existing studies support these findings, as associations between these mRNAs and LGG and other cancers have been reported. In terms of prognostic miRNAs, hsa-mir-421, has-mir-885, has-mir-495, hsa-mir-194-2, and hsa-mir-30d were identified as the most significant. These miRNAs also accord with existing studies that have reported associations between these miRNAs and tumors. Using the proposed Multi-PEN, it is expected that the estimation of both prognosis and prognostic genes can be performed. Furthermore, it is anticipated that future studies can expand the use of Multi-PEN to other cancer types.

However, there was a limitation in this study in that the validation dataset was not composed of fresh samples with independent datasets; instead, K-fold cross-validation was employed for the evaluation. While it is expected that this experimental setting with a single dataset using K-fold cross-validation properly evaluates the performance of the models, the validation with independent datasets can elaborate the experiments in a more precise manner. Additionally, the randomness of the weight parameter initialization in deep learning models produces moderately different results for each experimental test. Although this limitation was partially addressed by 100 repetitions of experiments and examining average performance, other deep learning techniques may be required to handle this problem fundamentally.

## Data Availability

The dataset employed in this study is a public dataset which is downloaded on 27 February 2022, received at Genomic Data Commons Data Portal: https://portal.gdc.cancer.gov/ (accessed on 30 August 2022).
